# New Insight into the Role of the Leucine Aminopeptidase 3 (*LAP3*) in Cell Proliferation and Myogenic Differentiation in Sheep Embryonic Myoblasts

**DOI:** 10.3390/genes13081438

**Published:** 2022-08-12

**Authors:** Ling Ge, Pengwei Su, Shan Wang, Yifei Gu, Xiukai Cao, Xiaoyang Lv, Shanhe Wang, Tesfaye Getachew, Joram M. Mwacharo, Aynalem Haile, Zehu Yuan, Wei Sun

**Affiliations:** 1 College of Animal Science and Technology, Yangzhou University, Yangzhou 225000, China; 2 International Joint Research Laboratory in Universities of Jiangsu Province of China for Domestic Animal Germplasm Resources and Genetic Improvement, Yangzhou 225009, China; 3 Joint International Research Laboratory of Agriculture and Agri-Product Safety of Ministry of Education, Yangzhou University, Yangzhou 225000, China; 4 International Centre for Agricultural Research in the Dry Areas, Addis Ababa 999047, Ethiopia

**Keywords:** sheep, *LAP3*, myoblast, proliferation, differentiation

## Abstract

Previous genome-wide association studies (GWAS) have found that *LAP3* may have the potential function to impact sheep muscle development. In order to further explore whether *LAP3* expression has an important role in the development of sheep embryonic myoblasts, we conducted the spatiotemporal expression profile analysis of *LAP3* at the tissue and cellular level. Then we used small interfering RNA and eukaryotic recombinant vectors to perform gain/loss-of-function analysis of *LAP3*. CCK-8 detection, EdU staining, and flow cytometry were used to investigate the impact of *LAP3* knockdown or overexpression on the proliferation of embryonic myoblasts. In addition, cell phenotype observation, MyHC indirect immunofluorescence, and quantitative detection of the expression changes of myogenic regulatory factors (MRFs) were used to explore the effect of *LAP3* on myogenic differentiation. The results showed that the *LAP3* expression level in muscle tissue of fetuses was significantly higher than that in newborn lambs and adult sheep, and its expression level on day 3 of differentiation was also significantly higher than that in the proliferation phase and other differentiation time points. *LAP3* silencing could significantly increase cell viability and EdU-positive cells, as well as prolonging the length of S phase of myoblasts to promote proliferation, while the results were reversed when *LAP3* was overexpressed. Moreover, *LAP3* silencing significantly hindered myotube formation and down-regulated the expression levels of MRFs from day 5 to day 7 of terminal differentiation, while the results were reversed when *LAP3* was highly expressed. Overall, our results suggested that the expression of *LAP3* impacts on the development of sheep embryonic myoblasts which provides an important theoretical basis for molecular breeding of meat production in sheep.

## 1. Introduction

Domestic sheep (*Ovis aries*) are one of the most promising types of livestock in global agriculture due to their advantages of fast growth, short reproductive cycle, and low disease risk. Maximizing their economic values to meet market demands is one of the main goals of sheep breeders [1]. Studies of sheep growth traits and genetic improvement, especially muscle growth and development, have become an essential part of sheep breeding. Finely identifying genomic regions and screening for genes associated with phenotypic traits of interest can provide candidates for gene marker-assisted selection (MAS) breeding [2].

In recent years, with advances in genome-wide association studies (GWAS), numerous studies have reported using GWAS to map candidate genes associated with muscle growth and development traits in sheep [3,4,5,6,7]. In a report of a multi-trait GWAS of 56 meat traits measured on 10,613 sheep [8], the leucine aminopeptidase 3 gene (*LAP3*) was located in the quantitative trait locus (QTL) region on chromosome 6 which was significantly associated with sheep growth and development traits. Meanwhile, La et al. found [9] that mutations in *LAP3* were traced in a Hu sheep population, affecting weight at different growth stages. From our previous study about expression quantitative trait loci (eQTL) analysis of sheep meat quality traits, we found that single nucleotide polymorphisms (SNPs) in the above QTL region were significantly correlated with gene expression [10]. Thus, we speculated that *LAP3*, as a potential candidate gene, plays a pivotal role in the sheep muscle growth and development process.

The evidence indicates that animals with a higher number of muscle fibers can provide a higher quantity and quality of meat, and the number of muscle fibers in mammals is mostly determined before birth, that is, during maternal pregnancy [11,12,13]. The prenatal developmental stage is directly related to the growth and development of individual skeletal muscles which determines the number of muscle fibers and postpartum muscle mass and further has a long-term impact on the production efficiency of animals after birth [14,15,16]. Embryonic skeletal muscle development mainly involves prenatal myogenesis, including myoblast proliferation, differentiation, and fusion to form multinucleated myotubes [17]. Briefly, myoblasts develop from mesenchymal stem cells of mesoderm origin. These progenitor cells enter the myogenic lineage, proliferate to establish a myoblast pool, then exit the cell cycle for differentiation, and finally fuse to form multinucleated myotubes [18]. To date, there are no reports on *LAP3* affecting embryonic myogenesis in sheep. Hence, we used sheep embryonic myoblasts as the experimental material for the gain/loss-of-function analysis of *LAP3* at the cellular level. 

In this study, our objective was to investigate whether *LAP3* affects the biological process of ovine myoblast proliferation and differentiation through a functional analysis at the cellular level. Here, we first used quantitative methods to analyze expression changes of *LAP3* at different growth stages and different differentiation stages of myoblasts in sheep. Then we knocked down and overexpressed *LAP3* to study the effect of *LAP3* on the proliferation of myoblasts. Finally, the induced differentiation model of myoblasts in vitro was established to study the effect of *LAP3* expression on the differentiation process of myoblasts. Collectively, all these results aim to gain insight into the role of *LAP3* in ovine muscle development and provide a rationale for sheep molecular breeding.

## 2. Materials and Methods

### 2.1. Ethical Statement

The animal experiment was reviewed and approved by the Experimental Animal Ethical Committee of Yangzhou University (license number 202103279, approved date: 2 March 2021). 

### 2.2. Sample Collection

The experimental Hu sheep were purchased from Suzhou Taihu Dongshan Sheep Industry Development Co., Ltd. (Suzhou 215000, Jiangsu Province, China). A total of six tissues; namely, heart, liver, spleen, lung, kidney, and *longissimus dorsi* muscle tissues were collected from three one-year-old adult sheep, three five-day-old newborn lambs, and three fetuses around day 85 after conception, respectively. The tissue samples were snap-frozen in liquid nitrogen and then immediately sent to the laboratory for storage at −80 °C until RNA extraction for spatiotemporal expression profile analysis of *LAP3*.

### 2.3. Isolation, Purification, and Identification of Embryonic Myoblasts

The fetal *longissimus dorsi* tissue used for the primary embryonic myoblasts’ isolation were obtained from the whole fetal sheep, placed in an insulated bucket, and immediately brought back to the laboratory without freezing. According to the previous description [19], the collagenase and trypsin combined digestion and differential adhesion methods were adopted. Firstly, the longissimus dorsi tissue was immersed in phosphate-buffered saline (PBS) containing 5% penicillin–streptomycin and then minced into small pieces around 1 mm^3^ with ophthalmic scissors. Secondly, tissue blocks were incubated at 37 °C with shaking (160 rpm) for 30 min in the presence of type IV collagenase (300 U/mL). The cell suspension was filtered through a 200-mesh cell sieve three times and then centrifuged at 1000 rpm for 5 min. Finally, the cell precipitation was resuspended with a high-glucose DMEM (growth medium, GM) containing 20% fetal bovine serum (FBS) and 2% penicillin–streptomycin and inoculated into a 60 mm petri dish at 37 °C, 5% CO_2_ incubator. Embryonic myoblasts were purified as described previously [20], adherent cells were mainly fibroblasts after the isolated primary muscle cells culturing for two hours, so the cell suspension that mainly contained myoblasts was re-inoculated into a 60 mm petri dish; this was repeated three times. Primary embryonic myoblasts were obtained after purification and were identified using quantitative analysis of MRFs after inducing differentiation. Then myoblasts with high differentiation potential were digested with 0.25% trypsin and frozen in liquid nitrogen for subsequent experiments.

### 2.4. Plasmid Construction and RNA Oligonucleotides

The coding sequence of *LAP3* was PCR-amplified from embryonic myoblasts complementary DNA (cDNA). The primers used are documented in Table 1. The linearized pcDNA3.1(+) vector was obtained by restriction endonuclease Hind III and BamH I digestion, and the PCR product (the target fragment) was inserted into the linearized vector according to the ClonExpress II One Step Cloning Kit instructions (Vazyme Biotech Co., Ltd., Nanjing, China). The successfully constructed recombinant plasmid was sent to Beijing Tsingke Biotechnology Co., Ltd. (Beijing, China) for verification and named pcDNA3.1(+)-LAP3 after confirmation sequencing. Small interfering RNA (siRNA) targeting ovine *LAP3* were synthesized by GenePharma Pharmaceutical Technology Co., Ltd. (Shanghai, China), together with a normal negative control (NC) without homology to the sequence of *LAP3*. Specific siRNA sequences are displayed in Table 2.

### 2.5. Cell Transfection and Induction of Differentiation

The transfection procedure was carried out when the cells grew to a confluence degree of 50–60% using the jetPRIME transfection reagent (Polyplus transfection, Strasbourg, Illkirch, France) according to the manufacturer’s instructions. The transfection groups were as follows: pcDNA3.1(+)/pcDNA3.1(+)-LAP3, siRNA-LAP3/NC, with three replicates per group. After 24–48 h transfection, embryonic myoblasts were induced to differentiate in vitro when GM was changed into a differential medium (DM, 98% high-glucose DMEM with 2% horse serum), with a replacement every two days. Cells were collected for subsequent RNA extraction.

### 2.6. Total RNA Extraction and Real-Time PCR (RT-PCR), and Reverse Transcription PCR

Total RNA was extracted from tissues and cells using the TRIzol reagent (TIANGEN, Beijing, China). The RNA concentration was measured with NanoReady spectrophotometer (Life Real, Hangzhou, China). RNA integrity and contamination were monitored on 1% agarose gels. Samples were stored in −80 °C until use. Reverse transcription was performed using FastKing gDNA Dispelling RT Super Mix (TIANGEN, Beijing, China). The reaction system was as follows: 5×FastKing-RT Super Mix 4 µL, total RNA 1000 ng, add RNase-free ddH_2_O to make the volume 20 µL. The reaction procedure was set as 42 °C for 15 min, 95 °C for 3 min and 4 °C indefinitely. RT-PCR was performed using 2×TSINGKE Master qPCR Mix (SYBR Green I) (Tsingke, Beijing, China). Each reaction volume contained 20 µL of 10 µL qPCR Mix, 0.8 µL each of 10 µM forward and reverse primers, 1 µL template and 7.4 µL sterile water. Amplification programs were 95 °C for 30 s, 95 °C for 10 s and 60 °C for 30 s, followed by 40 cycles using an CFX96 Connect™Real-Time System (BIO-RAD, Hercules, CA, USA). The mRNA expression level was normalized to the housekeeping gene *GAPDH* and analyzed using 2^−∆∆Ct^ method [21]. The primers used are displayed in Table 3.

### 2.7. Cell Counting Kit-8 (CCK-8) Assay

Cell proliferation ability was performed using CCK-8 analysis. In accordance with the CCK-8 kit instructions (Vazyme, Nanjing, China), the specific steps were as follows: embryonic myoblasts were seeded into the 96-well plates cultured in GM, and were transfected for 24–48 h, with six replicates per group. A 10 μL of cck-8 solution was added into each well gently to avoid bubbles, and cells were incubated at 37 °C in darkness for 2–3 h. Based on the fact that WST-8 could be reduced to highly water-soluble orange methylene dye (formazan) by some dehydrogenases in mitochondria under the action of electron coupling carrier 1-Methoxy PMS, the amount of formazan is proportional to the number of living cells. The optical density (OD) value at 450 nm was detected using a multifunction microplate reader (Tecan, Männedorf, Switzerland) at 0 h, 24 h, 48 h, and 72 h, which could indirectly reflect the relative cellular activity.

### 2.8. 5-Ethynyl-2′-deoxyuridine (EdU) Proliferation Assay

In accordance with the EdU cell proliferation (imaging test) kit instructions (RIBOBIO, Guangzhou, China), embryonic myoblasts were seeded into the 96-well plates cultured in GM, and were transfected for 24–48 h. Rapid detection of cell DNA replication activity based on the specific reaction of EdU and Apollo fluorescent dye in the kit could quickly and accurately detect cell proliferation ability. Fluorescent images were obtained from an inverted fluorescence microscope (Nikon, Minato, Tokyo, Japan). Image J (National Institutes of Health, Bethesda, MD, USA) software was used to count cell numbers in each image. The rate of EdU-positive cells was calculated with (EdU stained/Hoechst stained) × 100%.

### 2.9. Flow Cytometry for Cell Cycle Analysis

In accordance with the cell cycle kit instructions (Beyotime, Shanghai, China), embryonic myoblasts were seeded into the 6-well plates cultured in GM, and were transfected for 24–48 h. Briefly, the cells were digested by trypsin to be collected into a 1.5 mL centrifuge tube, then pre-cooled 70% ethanol was added to cells for fixation overnight at 4 °C. The propidium (PI) staining solution was prepared according to the instructions to dye the cell samples in darkness at 37 °C for 30 min. The DNA content of the cells was detected by the BD LSRFortessa flow cytometer (BD, Franklin Lakes, NJ, USA) after PI staining, and the distribution of DNA content was analyzed using Modfit (Version: 3.1) (Topsham, ME, USA).

### 2.10. Cell Immunofluorescence Assay

Cell differentiation was analyzed using immunofluorescence staining. Embryonic myoblasts were seeded into the 24-well plates cultured in GM, and were transfected for 24–48 h. When cells grew to 90% confluence, DM was added in cells instead of GM for 5–7 days. As described previously [22], the specific method was as follows: first, after removing the culture medium, PBS was used to wash the cells 2–3 times to remove excess dead cells, and 4% paraformaldehyde was added to incubate the cells for fixation at room temperature for 30 min, and PBS was used to wash the cells three times. Then, 0.5% TritonX-100 was added for cell penetration at room temperature for 20 min, and PBS was used to wash three times, and subsequently 5% goat serum was added to incubate at room temperature for 1 h. The cells incubated with primary antibodies anti-rabbit Myosin Heavy Chain (MyHC, 1:100, Affinity Bioscience, Cincinnati, OH, USA) overnight at 4 °C, washed with PBS for three times, then incubated with the second antibodies Goat Anti-Rabbit IgG (H+L) (1:200, Abcam, Cambridge Science Park, Cambridge, UK) in darkness for 2 h at room temperature. Finally, the cells were re-stained with 4′,6′-diamidino-2-phenylindole (DAPI, Vazyme) for 5–10 min at room temperature, and images were observed under inverted fluorescence microscope (Nikon, Minato, Tokyo, Japan).

### 2.11. Statistical Analysis

All the experimental results were presented as the means ± SEM (standard error of the mean). The normality of data distribution was verified by Kolmogorov–Smirnov Test using SPSS 25.0 software (SPSS, Inc., Chicago, IL, USA). Student’s *t*-test was used to determine if there were significant differences between the two sets of data, and one-way analysis of variance (ANOVA) followed by Duncan‘s multiple comparison test was used to compare significant differences between multiple sets of data. Differences among means with *p*-value < 0.05 was considered as statistically significant.

## 3. Results

### 3.1. Isolation, Culture, Induced Differentiation and Quantitative Identification of Sheep Embryonic Primary Myoblasts

**Figure 1:**
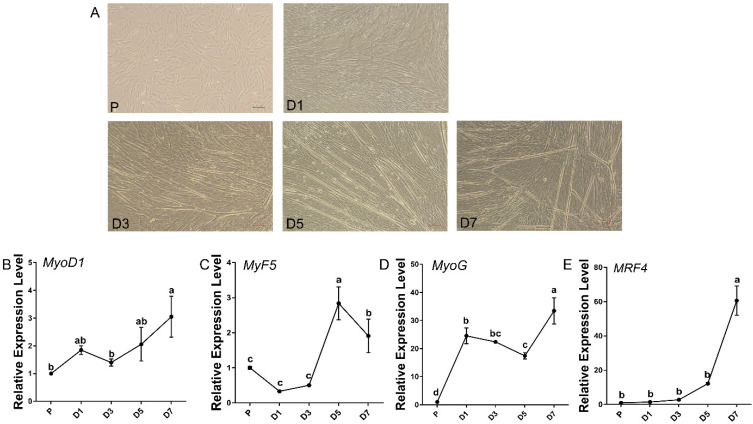
Isolation, culture, and differentiation of embryonic myoblasts and the expression analysis of MRFs genes at different differentiation stages. (**A**) The observation of embryonic myoblasts in bright field at proliferation (P) and the 1st, 3rd, 5th, and 7th day (D1, D3, D5, and D7) of differentiation using a fluorescence inverted microscope (100×, TS2R, Nikon). (**B**–**E**) The expression levels of *MyoD1* (**B**), *MyF5* (**C**), *MyoG* (**D**), and *MRF4* (**E**) mRNA were detected in embryonic myoblasts at proliferation (P) and the 1st, 3rd, 5th, and 7th day (D1, D3, D5, and D7) of differentiation by RT-PCR. Mean values with different letters were significantly different (*p* < 0.05) according to Duncan’s multiple range test; data are shown as mean ± SEM, *n* = 3 biological replicates.

According to the method for isolating myoblasts described in this study, we successfully isolated and purified sheep primary embryonic myoblasts. As shown in Figure 1A, the embryonic myoblasts cultured in GM grew well and adhered quickly, with a distinct long fusiform shape. When the cells grow before contact inhibition, that is, when the overall cell density occupies about 80–90% of the single-well culture area, we replaced the GM with DM to induced differentiation. On day 3 of induced differentiation, a large number of fused myotubes were formed (Figure 1A), indicating that the myoblasts had strong ability for differentiation.

Then, we quantitatively detected the expression levels of MRFs at different time points of differentiation. The expression levels of *MyoD1*, *MyoG*, and *MRF4* showed a steady upward trend (Figure 1B–E), among which the expression level of *Myf5* (Figure 1C) reached the highest on the day 5 of differentiation, and the expression level of *MRF4* peaked on day 7 of differentiation compared to other time points (*p* < 0.05). Our quantitative results were basically consistent with the expression patterns of MRFs reported in previous studies. The above results indicated that the embryonic myoblasts have the characteristics of high purity and high differentiation potential and can be used as the subsequent experimental material.

### 3.2. Expression Pattern Analysis of LAP3 at Different Stages of Sheep Muscle Development and Different Differentiation Stages of Embryonic Myoblasts

**Figure 2:**
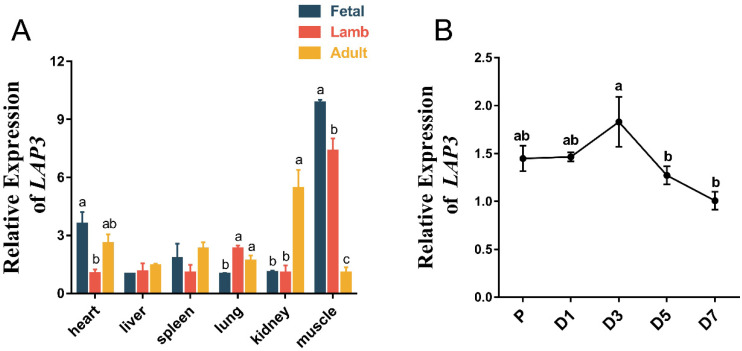
The expression levels of *LAP3* mRNA in different tissues at different growth stages and embryonic myoblasts at different differentiation stages. (**A**) The expression levels of *LAP3* mRNA were detected in the heart, liver, spleen, lung, kidney, and muscle tissues of fetal, lamb, and adult sheep by RT-PCR. (**B**) The expression levels of *LAP3* mRNA were detected of embryonic myoblasts at proliferation (P) and the 1st, 3rd, 5th, and 7th day (D1, D3, D5, and D7) of differentiation by RT-PCR. Mean values with different letters indicated that *LAP3* expression was significantly different at three development stages of fetal sheep, lambs, and adult sheep in the same tissue (*p* < 0.05) according to Duncan’s multiple range test; data are shown as mean ± SEM, *n* = 3 biological replicates.

We first analyzed the spatiotemporal expression profile of *LAP3* and used quantitative methods to detect the expression levels of *LAP3* in heart, liver, spleen, lung, kidney, and muscle tissues of fetal sheep, newborn lambs, and adult sheep. We found that *LAP3* was widely expressed in various tissues at different developmental stages, and the expression level of *LAP3* in muscle tissue of fetal sheep was significantly higher than those of newborn lambs and adult sheep (*p* < 0.05) (Figure 2A). From further expression detection at the cellular level, we found that expression level of *LAP3* on day 3 of inducted differentiation was significantly higher than those at other time points (*p* < 0.05) (Figure 2B). These results suggested that *LAP3* may act as an important role in sheep embryonic myoblasts.

### 3.3. Interfering with LAP3 Expression Promoted the Proliferation of Embryonic Myoblasts

**Figure 3:**
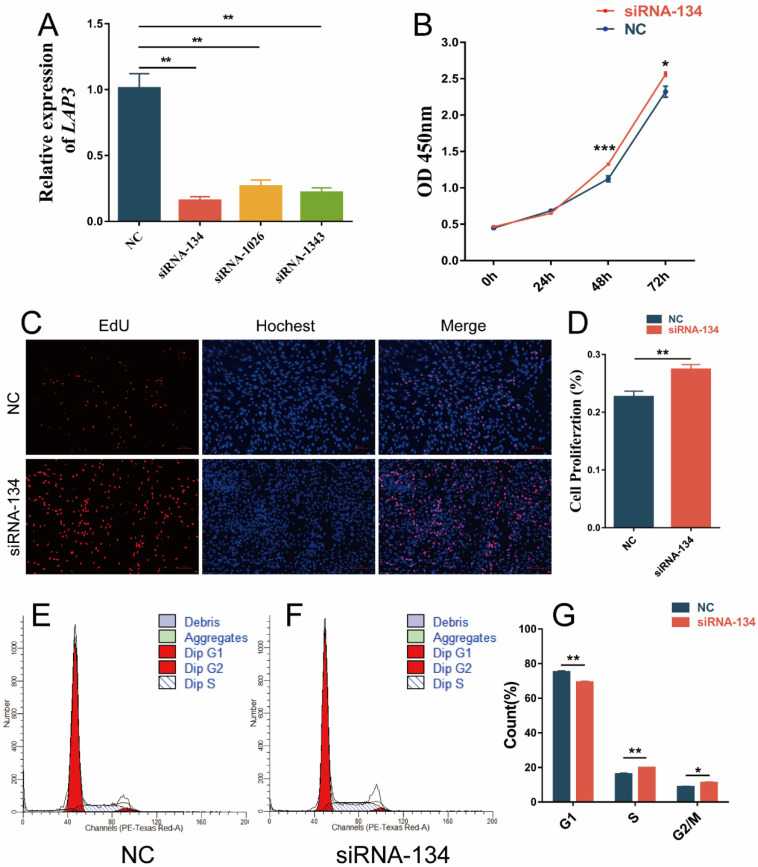
Effects of *LAP3* knockdown on embryonic myoblasts proliferation. (**A**) Interference efficiency of *LAP3* mRNA expression levels detected by RT-PCR in embryonic myoblasts transfected with siRNA-LAP3 or control. (**B**) CCK-8 assay of embryonic myoblasts transfected with siRNA-134 or control at 0 h, 24 h, 48 h, and 72 h. (**C**) EdU cell proliferation assay of embryonic myoblasts transfected with siRNA-134 or control (100×). (**D**) Cell proliferation rate of embryonic myoblasts transfected with siRNA-134 or control. (**E**,**F**) Flow cytometric measurement of DNA content using propidium iodide (PI) staining of embryonic myoblasts transfected with siRNA-134 or control. (**G**) Ratio of embryonic myoblasts at different stages of cell cycle transfected with siRNA-134 or control. * *p* < 0.05, significant difference; ** *p* < 0.01, or *** *p* < 0.001 extremely significant difference according to Student’s *t*-test; data are shown as mean ± SEM, *n* = 3 biological replicates.

In order to explore whether *LAP3* expression can affect the proliferation of embryonic myoblasts, we firstly examined three commercially available siRNAs. The quantitative results showed that the interference efficiency of siRNA-134 was the lowest (compared with the control group, fold change = 0.26) (*p* < 0.01) (Figure 3A), which showed that the silencing efficiency of *LAP3* expression reached more than 70%, so it could be used in the following experiments. However, siRNA-1026 and siNRA-1343 had a lower silencing efficiency, so they were not used in the subsequent experiments. CCK-8 detection of cell viability experiment showed that the OD values in *LAP3* interference group at 48 h and 72 h were significantly higher than those in the control group (*p* < 0.05 or *p* < 0.001) (Figure 3B), indicating that cell viability was up-regulated. The EdU staining showed that the ratio of EdU-positive cells in *LAP3* interference group was significantly higher than that in the control group (*p* < 0.01) (Figure 3C,D). The results of flow cytometry showed that compared with the control group, the S-phase progression in the *LAP3* interference group was significantly prolonged (*p* < 0.01) (Figure 3E–G).

### 3.4. Overexpression of LAP3 Inhibited the Proliferation of Embryonic Myoblasts

**Figure 4:**
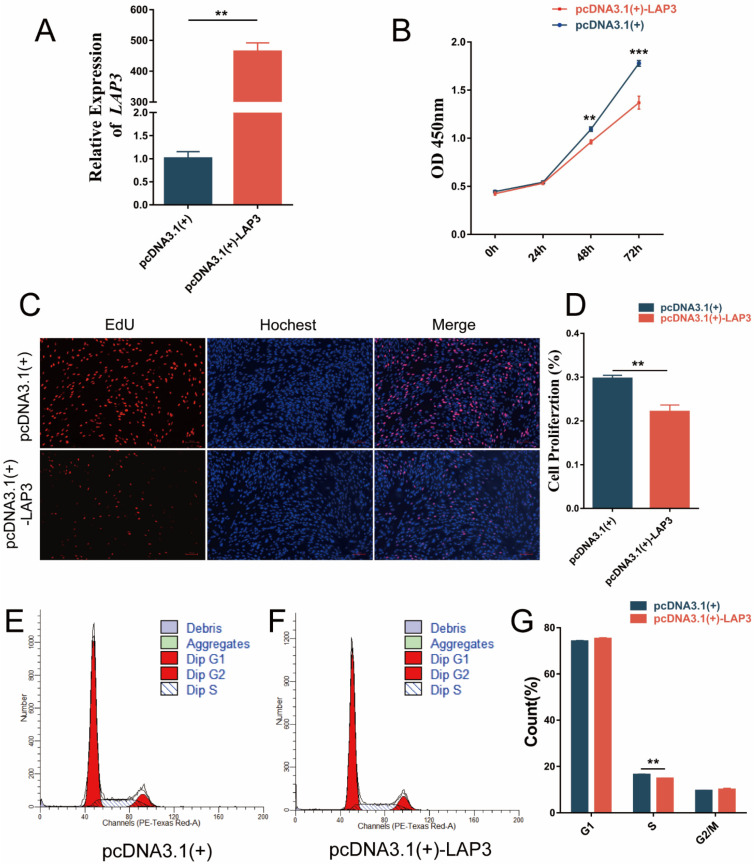
Effects of *LAP3* overexpression on embryonic myoblasts proliferation. (**A**) Overexpression efficiency of *LAP3* mRNA expression levels detected by RT-PCR in embryonic myoblasts. (**B**) CCK-8 assay of embryonic myoblasts transfected with pcDNA3.1(+)-LAP3 or pcDNA3.1(+) at 0 h, 24 h, 48 h, and 72 h. (**C**) EdU cell proliferation assay of embryonic myoblasts transfected with pcDNA3.1(+)-LAP3 or pcDNA3.1(+) (100×). (**D**) Cell proliferation rate of embryonic myoblasts transfected with pcDNA3.1(+)-LAP3 or pcDNA3.1(+). (**E**,**F**) Flow cytometric measurement of DNA content using propidium iodide (PI) staining of embryonic myoblasts transfected with pcDNA3.1(+)-LAP3 or pcDNA3.1(+). (**G**) Ratio of embryonic myoblasts at different stages of cell cycle transfected with pcDNA3.1(+)-LAP3 or pcDNA3.1(+). ** *p* < 0.01, or *** *p* < 0.001 extremely significant difference according to Student’s *t*-test; data are shown as mean ± SEM, *n* = 3 biological replicates.

We successfully constructed a *LAP3* overexpression recombinant vector, and the quantitative results showed that compared with the control group, the *LAP3* expression level was significantly increased (fold change = 464.52) (*p* < 0.01) (Figure 4A). CCK-8 detection showed that the OD values in *LAP3* overexpression group at 48 h and 72 h were significantly lower than those in the control group (*p* < 0.01 or *p* < 0.001) (Figure 4B), indicating that cell viability was down-regulated. The EdU staining showed that the ratio of EdU-positive cells in *LAP3* overexpression group was significantly decreased than those in the control group (*p* < 0.01) (Figure 4C,D). Flow cytometry showed that compared with the control group, the S-phase progression in *LAP3* overexpression group was significantly shorten (*p* < 0.01) (Figure 4E–G).

### 3.5. Interfering with LAP3 Expression Inhibits the Differentiation Process of Embryonic Myoblasts

**Figure 5:**
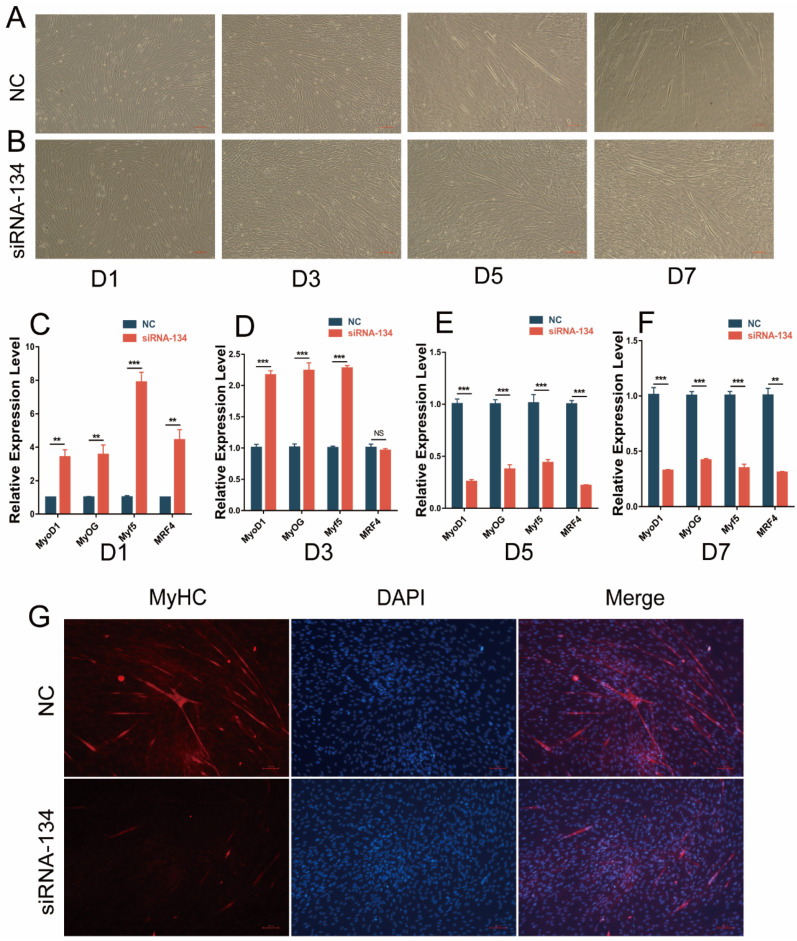
Effects of *LAP3* knockdown on embryonic myoblast differentiation. (**A**,**B**) The observation of embryonic myoblasts in bright field at 1st, 3rd, 5th, and 7th day (D1, D3, D5, and D7) of differentiation transfected with siRNA-LAP3 or control using a fluorescence inverted microscope (100×). (**C**–**F**) The expression levels of *MyoD1*, *MyF5*, *MyoG*, and *MRF4* in embryonic myoblasts transfected with siRNA-LAP3 or control at 1st (**C**), 3rd (**D**), 5th (**E**), and 7th (**F**) day (D1, D3, D5, and D7) of differentiation using RT-PCR. (**G**) Immunofluorescence staining of embryonic myoblasts transfected with siRNA-LAP3 or control using a fluorescence inverted microscope (100×). ** *p* < 0.01, or *** *p* < 0.001, extremely significant difference; ^NS^ *p* > 0.05, no difference according to Student’s *t*-test; data are shown as mean ± SEM, *n* = 3 biological replicates.

To further explore the impact of *LAP3* expression on the differentiation of embryonic myoblasts, we also performed gain/loss-of-function analysis. The cell phenotype observation of the induced differentiation model showed that the degree of myotube fusion in the *LAP3* interference group was lower than that in the control group (Figure 5A,B). The results of quantitative analysis showed that the expression levels of MRFs in *LAP3* interference group on day 5 and day 7 of induced differentiation were significantly lower than those in the control group (*p* < 0.01 or *p* < 0.001) (Figure 5E,F). The results of indirect immunofluorescence showed that MyHC immunostaining in the *LAP3* interference group decreased compared with the NC group on day 7 of differentiation (Figure 5G). All the above results suggested that *LAP3* silencing could affect the later differentiation of myoblasts.

### 3.6. Overexpression of LAP3 Promoted the Differentiation Process of Embryonic Myoblasts

**Figure 6:**
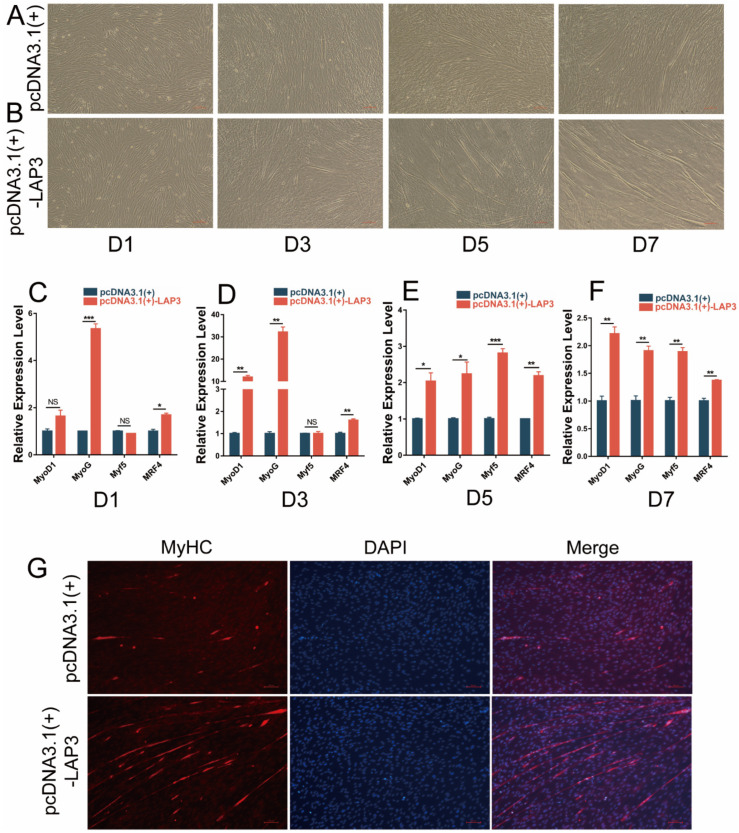
Effects of *LAP3* overexpression on embryonic myoblasts differentiation. (**A**,**B**) The observation of embryonic myoblasts in bright field at 1st, 3rd, 5th, and 7th day (D1, D3, D5, and D7) of differentiation transfected with pcDNA3.1(+)-LAP3 or pcDNA3.1(+) using a fluorescence inverted microscope (100×). (**C**–**F**) The expression levels of *MyoD1*, *MyF5*, *MyoG*, and *MRF4* in embryonic myoblasts transfected with pcDNA3.1(+)-LAP3 or pcDNA3.1(+) of at 1st (**C**), 3rd (**D**), 5th (**E**), and 7th (**F**) day (D1, D3, D5, and D7) of differentiation using RT-PCR. (**G**) Immunofluorescence staining of embryonic myoblasts transfected with pcDNA3.1(+)-LAP3 or pcDNA3.1(+) using a fluorescence inverted microscope (100×). * *p* < 0.05; ** *p* < 0.01, or *** *p* < 0.001, extremely significant difference; ^NS^ *p* > 0.05, no difference according to Student’s *t*-test; data are shown as mean ± SEM, *n* = 3 biological replicates.

Then we carried out the overexpression of *LAP3* to explore the effect on myoblast differentiation. The cell phenotype observation showed that the degree of myotube fusion in the *LAP3* overexpression group was higher than that in the control group (Figure 6A,B). Quantitative analysis showed that the expression levels of MRFs in the *LAP3* overexpression group on day 1 to day 7 of induced differentiation were significantly increased compared to those in the control group (*p* < 0.05, *p* < 0.01 or *p* < 0.001) (Figure 6C–F). Cell immunofluorescence assay showed that MyHC immunostaining in the *LAP3* overexpression group increased compared with the control group on day 7 of differentiation (Figure 6G). These results suggested that *LAP3* overexpression could promote the embryonic myoblasts.

## 4. Discussion

*LAP3* codes for leucine aminopeptidase 3, which is an important proteolytic enzyme that catalyzes the hydrolysis of leucine residues at the amino terminus of protein or peptide substrates, and is widely expressed in various tissues, such as muscle, liver, lens, kidney, among others [23,24]. An increasing number of GWAS reports in cattle have found that *LAP3* can be used as a candidate gene for traits such as caving [25], bone weight [26,27], and body size [28]. The potential of the *LAP3* gene has also attracted the attention of sheep breeders. Whether it is the GWAS of large populations and multiple traits or the detection of gene polymorphisms [8,9], the *LAP3* gene is also likely to be involved in sheep muscle development.

Therefore, we detected the *LAP3* expression levels in heart, liver, spleen, lung, kidney, and muscle tissues of fetuses, newborn lambs, and adult sheep to analyze the spatiotemporal expression profile. We found that *LAP3* expressed widely in all tissues, which is basically consistent with *LAP3* expression trends detected in these tissues of adult sheep by La et al. [9], and the expression level in muscle tissue of fetuses was significantly higher than those in newborn lambs and adult sheep. Based on this, we speculated that *LAP3* has an essential role in fetal skeletal muscle development. The fetal stage is thought to be the critical period for determining the number of muscle fibers which determines individual muscle mass and therefore impacts on animal productivity. A report investigating changes in tissue cellularity and metabolic assays at different phases of placental growth in sheep identified 70 to 80 days of gestation as the culmination of rapid fetal growth [29]. Faden et al. determined that around 85 days of gestation is the main stage of individual muscle fiber formation by observing the tissue sections of fetal muscles and quantitatively detecting the expression of factors such as IGF-II [30]. It is worth affirming that around 80 days of gestation is a rapid period of fetal muscle development from a recent proteomic analysis of sheep embryonic muscles [31].

In this study, we successfully isolated sheep embryonic myoblasts at 85 days of gestation, then established an in vitro differentiation model. We observed the formation of a large number of myotubes on the third day of induced differentiation, indicating that the myoblasts have relatively strong differentiation potential. Quantitative analysis showed that the expression levels of *MyoD1* gradually increased from the proliferation phase to the seventh day of induced differentiation. *MyoG* expression were at higher levels from day 3 to day 7 of induced differentiation. Meanwhile, the expression level of *MRF4* peaked on the seventh day of induced differentiation (compared to P, fold change = 60.6). The phenotypic assay of myogenic regulatory gene knockout mice and gene targeting assays have demonstrated that the activated expression of *Myf5* and *MyoD* during the proliferative phase of myoblasts are determinants of early myogenesis, and *MyoG* and *MRF4* act downstream, strictly control, and maintain terminal differentiation [32,33,34]. The MRF expression changes in this study were consistent with the reported expression patterns, indicating that the isolated sheep embryonic myoblasts were of high purity and could be used as subsequent experimental materials.

In order to explore whether the expression of *LAP3* affects the development of sheep embryonic myoblasts, quantitative results showed the expression level of *LAP3* on day 3 of differentiation was significantly higher than those in the proliferation phase and other differentiation time points. To further define the specific role of *LAP3* in myoblast proliferation and differentiation, we performed gain/loss-of-function analysis at the cellular level. After knocking down the *LAP3* expression, we found that *LAP3* silencing can significantly improve cell viability and prolong the length of S phase of myoblasts to promote proliferation. Then we carried out the *LAP3* overexpression study and found that *LAP3* overexpression significantly inhibited cell viability and shorten the length of S phase of myoblasts. The current functional studies on *LAP3* expression mainly focus on the field of cancer. In a study in which *LAP3* was knocked down or overexpressed in esophageal cancer cell lines, Li et al. found that *LAP3* overexpression facilitated esophageal cancer cells to overcome cell cycle arrest and promote proliferation [35], and the findings of another study in glioma cells were similar [36]. A possible explanation is that *LAP3* plays different but important roles in different cells and different biological processes.

It is well known that the myogenic differentiation of myoblasts can only be carried out after exiting the cell cycle. Proliferation and differentiation are two independent biological processes [37,38]. In order to further explore whether *LAP3* has an effect on myoblast differentiation, we used the in vitro differentiation model to knock down the *LAP3* expression, and found that myotube formation was significantly hindered in the *LAP3* interference group, and the expression levels of MRFs at day 5 and day 7 of terminal differentiation was significantly down-regulated. However, it was found that the formation of myotubes in the *LAP3* overexpression group was significantly promoted, and the expression levels of MRFs were significantly up-regulated to varying degrees throughout the differentiation stage. Collectively, we found that changes in the expression levels of *LAP3* could impact the differentiation process of sheep embryonic myoblasts. It is undeniable that the strong experimental evidence obtained in this study can be used as an important theoretical basis to support *LAP3* as an attractive molecular marker assists further sheep breeding.

**Figure 7:**
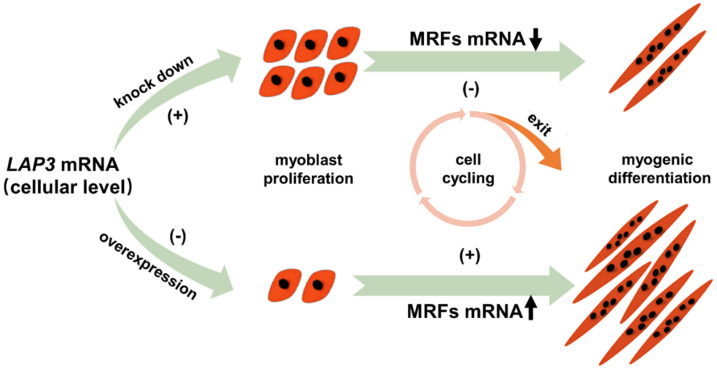
Proliferation and differentiation model of embryonic myoblasts after knockdown and overexpression of *LAP3* mRNA. (−) means inhibition; and (+) means promotion. The upper layer represented that knocking down of *LAP3* mRNA promoted myoblast proliferation, but down-regulated the expression levels of MRFs and inhibited myogenic differentiation of embryonic myoblasts; the lower layer represented that overexpression of *LAP3* mRNA inhibited myoblast proliferation, but up-regulated the expression levels of MRFs and promoted myogenic differentiation of embryonic myoblasts.

In summary, we took the growth and development of sheep muscle as the research background, and explored the impact of *LAP3* expression on the proliferation and differentiation of embryonic myoblasts from the perspective of embryonic myogenesis, which was shown in Figure 7. We have revealed the vital role of *LAP3*; namely, it negatively regulates myoblast proliferation and positively regulates myoblast differentiation, providing an important theoretical basis for *LAP3* to become a potential molecular marker, which is hopefully useful for molecular breeding of meat production as well as providing some new insights for future applications in sheep.

## 5. Conclusions

In conclusion, we revealed the vital role of *LAP3* expression on the proliferation and differentiation of embryonic myoblasts, which providing an important theoretical basis for *LAP3* to become a potential molecular marker for molecular breeding of meat production as well as providing some new insights for future applications in sheep.

## Figures and Tables

**Table 1:** Primers used in plasmid construction.

Name	Primer Name	Sequence (5′-3′)	Product Length (bp)
*LAP3*	*LAP3*-F	ATGTTCTTGCTGCCTCTTCCG	1560
	*LAP3*-R	CTAAGCACTGTCTTGACTGAACCGA	

**Table 2:** Sequence information of RNA oligonucleotides.

Name	Sequence Name	Sequence (5′-3′)
*LAP3*	siRNA-134	sense: GCGAGUAGCCGUCCGACAUTT
		antisense: AUGUCGGACGGCUACUCGCTT
	siRNA-1026	sense: GGAGCUGCCACUAUCUGUUTT
		antisense: AACAGAUAGUGGCAGCUCCTT
	siRNA-1343	sense: GCUGUGGAACAAACUAUUUTT
		antisense: AAAUAGUUUGUUCCACAGCTT

**Table 3:** Specific primers used for RT-PCR.

Name	Primer Name	Sequence (5′-3′)	GenBank Accession
*LAP3*	*LAP3*-F	ACGTCTTCATCAGACCCAAGT	XM_012179698.4
	*LAP3*-R	AGCCTTGATGGAGATGCCAC	
*MyoD1*	*MyoD1*-F	AACTGTTCCGACGGCATGAT	NM_001009390.1
	*MyoD1*-R	TGTAGTAAGCGCGGTCGTAG	
*Myf5*	*Myf5*-F	CCTCAAGTTGCTCTGATGGC	XM_015094556.3
	*Myf5*-R	ATCCAGGTTGCTCTGAGTTGG	
*MyoG*	*MyoG*-F	CTCAACCAGGAGGAGCGTGAT	NM_001174109.1
	*MyoG*-R	GTGGGCATCTGTAGGGTCCG	
*MRF4*	*MRF4*-F	GCTACAGACCCAAGCAGGAA	NM_001134782.1
	*MRF4*-R	CGAGGCCGATGAATCAATGC	
*GAPDH*	*GAPDH*-F	TCTCAAGGGCATTCTAGGCTAC	NM_001190390.1
	*GAPDH*-R	GCCGAATTCATTGTCGTACCAG	

## Data Availability

Not applicable.
